# Empirical Bayes single nucleotide variant-calling for next-generation sequencing data

**DOI:** 10.1038/s41598-024-51958-z

**Published:** 2024-01-18

**Authors:** Ali Karimnezhad, Theodore J. Perkins

**Affiliations:** 1 Department of Mathematics and Statistics, University of Ottawa, Ottawa, K1N 9A7 Canada; 2 Biostatistics and Risk Modelling Division, Bureau of Food Surveillance and Science Integration, Food Directorate, Health Products and Food Branch, Health Canada, Ottawa, K1A 0K9 Canada; 3 Regenerative Medicine Program, Ottawa Hospital Research Institute, Ottawa, K1H 8L6 Canada; 4 Department of Biochemistry, Microbiology and Immunology, University of Ottawa, Ottawa, K1H 8M5 Canada

**Keywords:** Statistics, Cancer genomics

## Abstract

One of the fundamental computational problems in cancer genomics is the identification of single nucleotide variants (SNVs) from DNA sequencing data. Many statistical models and software implementations for SNV calling have been developed in the literature, yet, they still disagree widely on real datasets. Based on an empirical Bayesian approach, we introduce a local false discovery rate (LFDR) estimator for germline SNV calling. Our approach learns model parameters without prior information, and simultaneously accounts for information across all sites in the genomic regions of interest. We also propose another LFDR-based algorithm that reliably prioritizes a given list of mutations called by any other variant-calling algorithm. We use a suite of gold-standard cell line data to compare our LFDR approach against a collection of widely used, state of the art programs. We find that our LFDR approach approximately matches or exceeds the performance of all of these programs, despite some very large differences among them. Furthermore, when prioritizing other algorithms’ calls by our LFDR score, we find that by manipulating the type I-type II tradeoff we can select subsets of variant calls with minimal loss of sensitivity but dramatic increases in precision.

## Introduction

Recent developments in next generation sequencing (NGS) technologies provide an insight into the task of mutation calling^1^ and have made it possible to characterize the genomic alterations in a tumor in an unbiased manner^2^. With the advancement of NGS technologies, the number of large-scale projects (especially cancer projects) dealing with point mutation in various tumor types has been increased rapidly, and many bioinformatics tools have been developed.

Several packages with different algorithms have been introduced in recent years to increase the accuracy in the mutation detection procedure. Perhaps, SAMtools^3^ is the most cited package for manipulating and converting alignments to different formats. It is also used for sorting and merging alignments, generating per-position information in pileup and mpileup formats, as well as calling single nucleotide variants (SNVs) and short insertion and deletions (INDELS). VarScan2^4^, a newer version of VarScan^5^, reads SAMtools’s pileup or mpileup output and detects SNVs, INDELs and copy number variations using separate commands. Mutect^6^, an algorithm based on a Bayesian classifier, is claimed to be highly sensitive in the detection of very low frequency SNVs. In this paper, we use the Mutect2 tool in GATK that was developed by the Broad Institute and is used widely in the context of mutation-calling. VarDict^7^ calls different types of mutations, including SNVs, multiple-nucleotide variants, INDELs, complex and structural variants at the same time. Pisces^8^, which includes a variant-collapsing algorithm to unify variants broken up by read boundaries, basic filtering algorithms, and a simple Poisson-based variant confidence-scoring algorithm, is aimed to reduce noise or increase the likelihood of detecting true variants. In a recent study, Karimnezhad et al.^9^ compared the performance of the above-mentioned variant callers on different sets of NGS datasets sequenced on different platforms. For a list of recent mutation callers, readers may also refer to Xu^10^, where 46 mutation callers are reviewed.

Most variant callers in the literature rely on several pre-defined but adjustable parameters such as minimum base call quality (*BQ*), minimum mapping quality (*MQ*), strand bias threshold, etc. Remarkably, many of the parameters as well as their default values are not the same among the existing variant callers. For example, the default value for minimum allele frequency (AF) in Mutect2 is set to 0. See Table 1 for a list of selected parameters along with their default values in some selected algorithms. These parameters can strongly affect the output of the variant-calling algorithms. Best values for the parameters may in general depend on the type of sequencing data, the type of mutations sought (somatic or germline), noise characteristics of the dataset, etc. As such, most users/analysts may not have enough expertise to adjust those pre-determined parameters properly. On the other hand, relying on default values for parameters may result in unreliable results and is problematic when comparing programs, because their default parameters differ.

**Table 1:** Default values for options in different mutation callers.

Option	Mutect2	VarScan2	SAMtools	VarDict	Pisces
Threshold for AF	0	0.01	–	0.05	0.01
Min *BQ* score	10	15	13	22.5(Illumina)	20
				15(PGM)	
*BQ* score threshold	18	–	–	–	—
Max *BQ* score	–	–	–	–	100
Min *MQ*	20	null	0	null	1
Mean min *MQ*	–	null	–	null	–
Maximum min *MQ*	null	–	–	–	–
Min coverage	–	8	–	–	10
Maximum coverage	–	–	250	–	–
Supporting reads to call a variant	–	2	–	–	–
Minimum variant quality score	–	–	–	–	15
Threshold for variant quality score filter	–	–	–	–	20
Strand bias filter	–	–	–	–	0.5
Minimum reads to strand bias	2	–	–	–	–

A dash means that the corresponding parameter was not defined in the caller’s settings.

The above approaches are either conventional Bayesian or frequentist methods, and do not take multiplicity and testing efficiency issues into account. Moreover, many programs fail to output a criterion by which SNV calls can be ranked, such as p-values, so that adjusting preference between type I and type II errors or constructing ROC curves is not naturally supported.

Some studies in the literature proposed germline variant detection in multi-sample NGS data using Bayesian methods. For example, Huang et al.^11^ raised the question of how to quantify sequencing errors. They argue that it may not be ideal to quantify sequencing error only based on quality scores and propose an empirical Bayes method based on a Binomial model to estimate sequencing errors and identify variants. In another study, Zhao et. al^12^ developed an optimal empirical Bayesian testing procedure to detect variants in NGS data, which is based on pooling a normalized amount of DNA from multiple samples. Due to a capacity issue, they assume samples may be distributed and sequenced independently in $M>1$ pools and each pool consists of *N* individuals. Although having *M* pools with *N* individuals may be cost-effective, however, our available samples (similar to many other clinical labs) instruct us to base the model only on one DNA sample for one individual. Similar to Huang et al.^11^, Ding et al.^13^ suggested that estimating sequencing errors based on quality scores may not be precise. Using single-sample NGS data, they proposed an empirical Bayes method to model the dispersion of minor AF and call germline variants.

In this paper, we propose a Binomial model and include an error rate based on a weighted average of base call and mapping quality scores as part of model parameters. We use an empirical Bayesian approach to develop a local false discovery rate (LFDR) estimator for SNV detection. In contrast to existing algorithms, our novel approach calls SNVs not just on a site-by-site basis, but by simultaneously using information across all the sites to build a probabilistic model of the data.

To the best of our knowledge, LFDR estimation has not been employed in the task of variant-calling, but it has been well developed in a variety of other contexts, and different strategies have been introduced in the literature. To name a few LFDR estimation based approaches, readers may refer to Pan et al.^14^, Efron et. al^15^, Efron^16^, Padilla and Bickel^17^, Yang et al.^18^, Karimnezhad and Bickel^19^, and Karimnezhad^20^.

Obviously, all variant-calling algorithms determine mutations based on some observed evidence, but since evidence supporting variants (including number of reference and alternative read counts) differs from site to site, the confidence in calling a site as a variant site varies. We fill this gap by introducing an LFDR-based algorithm that meaningfully scores variants called by any variant caller and prioritizes them from most to least probable variants. This helps with significantly reducing many false positives.

The structure of this work is as follows. In the “Methods” section, we provide a detailed presentation of the model, method and the algorithm we propose for germline SNV calling. We also propose a modified version of the LFDR algorithm so that it can prioritize variants called by any desired variant caller. In the “Results” section, we use a suite of gold-standard cell line data to evaluate the performance of our proposed LFDR approach against a collection of widely used, state of the art programs: MuTect2, SAMtools, VarScan2, VarDict, and Pisces.

## Methods

We consider analyzing a single DNA sample from a healthy patient, where only germline mutations are expected to be seen. We also assume that the data has been mapped to a reference genome, which specifies one of *A*, *C*, *G*, or *T* bases for every position. Each mapped read shows one of the four possible bases (*A*, *C*, *G*, or *T*) for the same position. With no loss of generality, we also suppose, for a replicate (technical or biological), that sequencing covers *p* sites (individual positions in the genome) of which $p_{0}$ sites are non-mutant. For each locus *i*, $i=1,\ldots ,p$, we assume that there are $K_{i}$ (short) known number of reads covering the locus *i*. At each locus *i*, we suppose there are four possible bases (*A*, *C*, *G*, *T*) of which $R_i$ random reads carry the reference allele. We further suppose that of the remaining $K_i-R_i$ reads, $M_{i}$ random number of reads carry the most alternative (dominant) allele and, we assign the remaining random alternative reads to $X_{1i}$ and $X_{2i}$. We briefly refer to these random variables by ${\varvec{X}}_i=(R_{i},M_{i},X_{1i},X_{2i})$. Generally, the larger $M_i$ is, the more support we have for an alternative allele being present at site *i* in the DNA being sequenced. Indeed, if the data had no sequencing or mapping errors, $M_i>0$ could only arise as the result of an alternative allele being present. However, it is well-recognized that sequence data does contain errors. Although those errors can arise in many ways, the standard model error employed by the community is the error in mistakenly reporting an alternative allele as one of the other three possible alleles, and vice versa. We refer to this error by $e_i$. Obviously, if there is no error, mutant sites are expected to be those $p-p_{0}$ sites, for which $M_i$ is positive.

Suppose that $\theta _{i}$ represents the dominant alternative AF at the *i*th site. Obviously, given the information that *i*th site has no mutation, $\theta _{i}=0$ and otherwise, $\theta _{i}>0$. We assume $P(\theta _i=0)=\pi _0$ and $P(\theta _i>0)=1-\pi _0$. If $\theta _i>0$, we assume it follows a probability density function $g(\cdot )$. We assume that each read is drawn independently from either the reference or alternative allele pools. However, we also assume an independent chance $e_i$ of read error in each read at each site, which randomly changes the correct allele to one of the other three options. The error term $e_i$ can be calculated in different ways. For example, in Mutect, error is estimated based on Phred-scaled quality scores of mapped reads to each position. We suggest to take the error rate as a weighted average of average base call qualities and average mapping qualities. Supposed at position *i*, for an arbitrary base pair $U\in \{A,C,G,T\}$, $BQ_{i}^U$ and $MQ_{i}^U$ represent average base call quality and average mapping quality across all reads at that position, respectively, and let $Q_i^U$ be the average of $BQ_{i}^U$ and $MQ_{i}^U$, i.e., $Q_{i}^U=\frac{1}{2}(BQ_{i}^U+MQ_{i}^U)$. Depending on whether the base pair observed at position *i* is a reference (*R*), dominant (*M*) or one of the remaining alternative alleles ($X_1$ or $X_2$), we calculate $Q_i^R$, $Q_i^M$, $Q_i^{X_1}$ and $Q_i^{X_2}$, and use them to calculate the weighted average quality $Q_i=\frac{Q_i^R\times R_i+Q_i^M\times M_i + Q_i^{X_1}\times X_{1i} + Q_i^{X_2}\times X_{2i}}{R_i+M_i+X_{1i}+X_{2i}}$. Then, we define the error rate to be $e_i=10^{-\frac{Q_i}{10}}$. As in Mutect^6^, we define the following four probabilities. The probability that a randomly chosen read covering site *i* shows the reference allele is $p_{R_i}=\theta _i\frac{e_i}{3}+(1-\theta _i)(1-e_i)$. This is explained as the sum of the probabilities that the DNA actually contained the reference allele at site *i* and it was correctly read, $(1-\theta _i)(1-e_i)$, and probability that the DNA actually contained the alternative allele but it was misread, and by chance, it was misread as the reference allele, $\theta _i\frac{e_i}{3}$. Similarly, the probability that a random read covering site *i* shows the alternative allele is $p_{M_i}=\theta _i(1-e_i)+(1-\theta _i)\frac{e_i}{3}$, which arises either as correct reading of the alternative allele or misreading the reference allele as the alternative. Finally $p_{X_{1i}}=p_{X_{2i}}=\frac{e_i}{3}$ is the chance that one of the two other alleles that are not reference or alternative occurs at site *i*. Putting these together, the probability of the total data at site *i*, ${\varvec{X}}_i$ is multinomial with $K_i$ tries (reads) and probabilities of reference, alternative, and the other two alleles. At this point, we diverge from the model of Mutect by proposing a new model governing the parameter $\theta _i$, which ultimately allows us to develop our empirical Bayesian LFDR approach. We assume ${\varvec{X}_i}|\theta _{i}\sim \text {Multi}\left( K_{i},p_{R_i},p_{M_i},p_{X_{1i}},p_{X_{2i}}\right)$ where $p_{R_i}=\theta _{i}\frac{e_i}{3}+(1-\theta _{i})(1-e_i)$, $p_{M_i}=\theta _{i}(1-e_i)+(1-\theta _{i})\frac{e_i}{3}$, $p_{X_{1i}}=\frac{e_i}{3}$, $p_{X_{2i}}=\frac{e_i}{3}$, where $\theta _i$ could be either 0 or positive with probabilities $\pi _0$ and $1-\pi _0$, respectively. If $\theta _i>0$, we assume $\theta _i\sim g(\cdot )$, where $g(\cdot )$ is unknown. In our proposed approach we do not need to know the form of $g(\cdot )$.

To discover whether site *i* is a mutant site, we propose testing the null hypothesis $H_{0i}:\theta _i=0$ against the alternative hypothesis $H_{1i}:\theta _i>0$, $i=1,\ldots ,p$. To do so, we focus on estimating $\psi _{i}\equiv P(\theta _i|{\varvec{X}}_i)$, the posterior probability that the null hypothesis $H_{0i}$ is true. Once estimated, it is compared with a pre-specified threshold leading to either rejecting or failing to reject the null hypothesis. The quantity $\psi _{i}$ is well-known in the literature as LFDR.

With the above settings, the following probability functions are derived under the null and the alternative hypotheses, respectively,

1 $$\begin{aligned} P({\varvec{X}}_i | \theta _i=0) = {K_{i} \atopwithdelims ()R_{i},M_{i},X_{1i},X_{2i}}\left( 1-e_i\right) ^{R_{i}}\left( \frac{e_{i}}{3}\right) ^{M_{i}+X_{1i}+X_{2i}}= {K_{i} \atopwithdelims ()R_{i},M_{i},X_{1i},X_{2i}}\left( 1-e_i\right) ^{R_{i}}\left( \frac{e_{i}}{3}\right) ^{K_i-R_i} \end{aligned}$$

and

2 $$\begin{aligned} P({\varvec{X}}_i | \theta _i>0)&=\int P({\varvec{X}}_i | \theta _i>0)g(\theta _{i})d\theta _{i}\nonumber \\&=\int _{0}^{1}{K_{i} \atopwithdelims ()R_{i},M_{i},X_{1i},X_{2i}}\left( \theta _{i}\frac{e_i}{3}+(1-\theta _{i})(1-e_i)\right) ^{R_{i}}\left( \theta _{i}(1-e_i)+(1-\theta _{i})\frac{e_i}{3})\right) ^{M_{i}}\left( \frac{e_i}{3}\right) ^{X_{1i}+X_{2i}}g(\theta _{i})d\theta _{i}. \end{aligned}$$

Now, using the Bayes formula, the LFDR can be expressed by

3 $$\begin{aligned} \psi _{i}=\frac{\pi _{0} P({\varvec{X}}_i | \theta _i=0)}{\pi _{0}P({\varvec{X}}_i | \theta _i=0) +(1-\pi _{0})P({\varvec{X}}_i | \theta _i>0)}, \end{aligned}$$

where $P({\varvec{X}}_i | \theta _i=0)$ and $P({\varvec{X}}_i | \theta _i>0)$ are given by (1) and (2), respectively, and $\pi _0$ is the proportion of non-mutant sites. Both the parameters $\pi _0$ and $g(\theta _{i})$ (in $P( {\varvec{X}}_i | \theta _i>0)$) are unknown and need to be estimated before making any inference.

Now, let $\delta _{i}$ be a binary decision rule corresponding to *i*th set of hypotheses $H_{0i}$ and $H_{1i}$. We assume that $\delta _{i}=1$ if the null hypothesis $H_{0i}$ is rejected, and $\delta _{i}=0$, otherwise. But since such binary decisions can lead to some errors, we define the following loss function when testing the null hypothesis at site *i*:

$$\begin{aligned} L(\theta _{i},\delta _{i})={\left\{ \begin{array}{ll} 0 &{} \theta _i=0,\,\delta _{i}=0\,\,\text {or}\,\theta _i>0,\,\delta _{i}=1,\\ l_{I} &{} \theta _i=0,\,\delta _{i}=1,\\ l_{II} &{} \theta _i>0,\,\delta _{i}=0, \end{array}\right. } \end{aligned}$$

where $l_{I}$ and $l_{II}$ are loss values incurred due to making type I and type II errors, respectively, see Karimnezhad and Bickel^19^. Also, see Zhao et. al^12^ where a specific version of this loss with $l_I=\lambda$ and $l_{II}=1$ is used.

To take all *p* sites in a sample to account, suppose that $\varvec{\delta }=(\delta _{1},\ldots ,\delta _{p})$ represents a vector of estimators of $\varvec{\theta }=(\theta _{1},\ldots ,\theta _{p})$ and measure the aggregated loss by $L(\varvec{\theta },\varvec{\delta })=\sum _{i=1}^{p}L(\theta _{i},\delta _{i})$, which takes both type-I and type-II errors into account. Now, to derive a Bayesian decision rule in the above-mentioned hypothesis testing problem, let $\rho _{\varvec{X}}(\varvec{\theta },\varvec{\delta })=E[L(\varvec{\theta },\varvec{\delta })|\varvec{X}]$ denote the posterior risk of choosing a decision vector $\varvec{\delta }$, where $\varvec{X}=(\varvec{X}_1,\ldots ,\varvec{X}_p)$. Then, similar to Karimnezhad and Bickel^19^, it can be verified that the Bayesian decision vector $\varvec{\delta }^{B}=(\delta _{1}^{B},\ldots ,\delta _{p}^{B})$ with

4 $$\begin{aligned} \delta _{i}^{B}={\left\{ \begin{array}{ll} 0 &{} \psi _{i}>\frac{l_{I}}{l_{I}+l_{II}},\\ 1 &{} \psi _{i}\le \frac{l_{I}}{l_{I}+l_{II}},\\ \end{array}\right. } \end{aligned}$$

where $\psi _{i}$ is given by (3), minimizes the posterior risk w.r.t. $\varvec{\delta }$.

The above equation suggests that mutant sites can be determined by estimating $\psi _i$, $i=1,\ldots ,p$, and then comparing it with the threshold $\frac{l_{I}}{l_{I}+l_{II}}$. However, estimating $\psi _i$ is challenging due to the complicatedness of the form of $P({\varvec{X}}_i | \theta _{i}>0)$ as well as $g(\cdot )$, the distribution of AFs.

**Figure 1:**
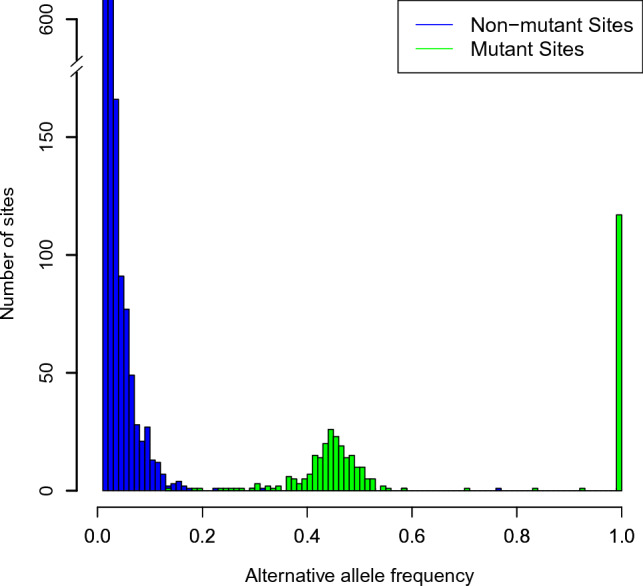
Histogram of distribution of alternative AFs from TST-GM12878 data.

One immediate solution to estimating $g(\cdot )$ would be to assign a non-informative prior to the alternative AFs $\theta _{i}$. In a similar situation, Zhao et. al^12^ consider a uniform distribution on the interval (0, *a*), and then they estimate the value of *a* as well as $\pi _0$ using the traditional method of moments. While simple, this assumption seems unrealistic. Figure 1 represents the distribution of AFs at known mutant as well as non-mutant sites from TST-GM12878 data (see Results for more information).

From Fig. 1, we observe that the assumption that AFs take any number between 0 and 1 with equal probability is unrealistic. Estimating $\pi _0$ may be connected to the estimation of $g(\cdot )$ and consequently, a bad estimate of $g(\cdot )$ may directly lead to a bad estimate of $\pi _0$. This indeed is true in some datasets with ambiguous AF ranges, including our datasets. Clearly, from this figure, AFs of mutant sites is distributed around either 0.5 or 1. This highlights the importance of finding a suitable estimate of $g(\cdot )$. Dependency of estimated values of parameters of LFDR to each other can be seen in different algorithms in the literature. See for example Zhao et. al^12^ where their proposed estimator of $\pi _0$ is a function of the estimated value of the upper bound *a* in the uniform prior assigned to the alternative AF. For other examples, see Pan et al.^14^ and Karimnezhad and Bickel^19^.

As Fig. 1 suggests, one may fit a beta distribution to model the alternative AFs, which adds extra parameters to the model. To avoid model complexity, as an innovative approach, we suggest estimating $g(\cdot )$ empirically, based on the alternative AFs at a set of known or suspected mutated sites. This raises the question of which set of sites to use.

An immediate solution to the above problem would be to take all sites for which $M_{i}$ counts are positive. But as many non-zero alternative AFs in NGS studies turn out to correspond to artifacts, we need to find and exclude them from our list so that a precise empirical distribution for $g(\cdot )$ can be estimated. Suppose that ${\mathscr {I}}_s$ stands for the set of *s* indices of those non-zero $\theta _{i}$ corresponding to mutant sites, and let $\widehat{\varvec{\theta }}_s$ be the vector of the corresponding empirical AFs. In our settings, *s* represents the number of elements of the set ${\mathscr {I}}_s$. Now, let $G(\cdot )$ represent the cumulative distribution function (CDF) that corresponds to $g(\cdot )$. Then, the corresponding empirical CDF is given by $\widehat{G}_s(t)=\frac{1}{s}\sum _{l\in {\mathscr {I}}_s}1_{\theta _l\le t}$, $t\in \Re$. Obviously, due to the strong law of large numbers, $\widehat{G}_s(t)$ converges to *G*(*t*) as $n\rightarrow \infty$ almost surely, for every value of *t*, and this implies that $\widehat{G}_s(t)$ is a consistent estimator. Then, one may estimate $P({\varvec{X}}_i | \theta _i>0)$ by

5 $$\begin{aligned} P_{\widehat{\varvec{\theta }}_s}({\varvec{X}}_i | \theta _i>0)&\simeq \frac{1}{s}\sum _{l\in {\mathscr {I}}_s}\left\{ {K_{i} \atopwithdelims ()R_{i},M_{i},X_{1i},X_{2i}}\left( \theta _{l}\frac{e_i}{3}+(1-\theta _{l})(1-e_i)\right) ^{R_{i}}\left( \theta _{l}(1-e_i)+(1-\theta _{l})\frac{e_i}{3})\right) ^{M_{i}}\left( \frac{e_i}{3}\right) ^{X_{1i}+X_{2i}}\right\} . \end{aligned}$$

Since ${\mathscr {I}}_s$ includes many artifacts, *s* needs to be optimally learned. We propose an algorithm that allows for repeatedly updating the set of mutant sites and leads to a precise estimate of $g(\cdot )$.

### An empirical Bayes mutation detection procedure

To discover mutant sites, we propose the procedure outlined in Algorithm 1. The proposed algorithm, like many existing procedures in the literature, including Mutect2, VarScan2^4^, VarDict^7^ and Pisces^8^ is comprised of three main steps.

In the pre-processing step, we propose excluding low quality bases from the input file (either FASTQ or BAM format). Thus, observations in our model are only those bases that pass a minimum average *BQ* and average *MQ* threshold. *BQ* corresponds to an error rate of $e=10^{-\frac{BQ}{10}}$, see for example Cibulskis et al.^6^ and Dunn, et al.^8^. Thus taking $BQ=20$ corresponds to an error rate of 0.01 which can be inferred as expecting one miscalled base in reading 100 bases. *MQ* is related to aligning reads to a reference genome, and it usually varies between 0 and 60. Similar to Pisces, we impose a threshold of 20, and 30 to the minimum *BQ* and *MQ*, respectively. Different publicly available softwares such as SAMtools^3^ and bam-readcount (https://github.com/genome/bam-readcount) can be applied to exclude such low quality reads. Once low quality bases are excluded, the counts $K_{i}$, $R_{i}$, $M_{i}$, $X_{1i}$ and $X_{2i}$ need to be formed.

Although in Eq. (5) we only include all non-zero empirical AFs, one may tweak the algorithm by just assuming that AFs follow, for example, a uniform distribution on the interval (0, 1), or a subset of it. Thus, ${\mathscr {I}}_s^j$ in Step 2.6 of Algorithm 1, may be alternatively taken to be a set of *N* (for example 1000) sites for which their AFs are randomly generated from a uniform distribution on the interval (0, 1). However, once the estimation procedure enters Step 2.10, the updated ${\mathscr {I}}_s^j$ set will only depend on the actual empirical AFs. A third approach would be to estimate LFDRs by focusing only on the original uniformly sampled AFs, without updating the set of indices ${\mathscr {I}}_s^j$ in step 2.10, i.e., for all *j*, ${\mathscr {I}}_s^j={\mathscr {I}}_s^1$. We refer to this approach by “uniform” estimation. For comparison purposes, we use these three methods in our data analysis and will refer to them by “empirical”, “uniform/empirical” and “uniform” estimation of $g(\cdot )$, respectively. In Step 2.10, we took $\epsilon =0.001$.

In the post-processing step, we take 0.01 and 10 as default AF threshold (*AFT*) and read depth threshold (*DPT*) values, respectively. This is not uncommon, and many variant callers impose such thresholds. For example, Pisces^8^ has the same default values for these thresholds. We remark that one may apply this filtering in Step 1. However, the final estimated $\pi _0$ will reflect the proportion of non-mutant sites w.r.t. the filtered input file rather than the original one.

**Algorithm 1:**
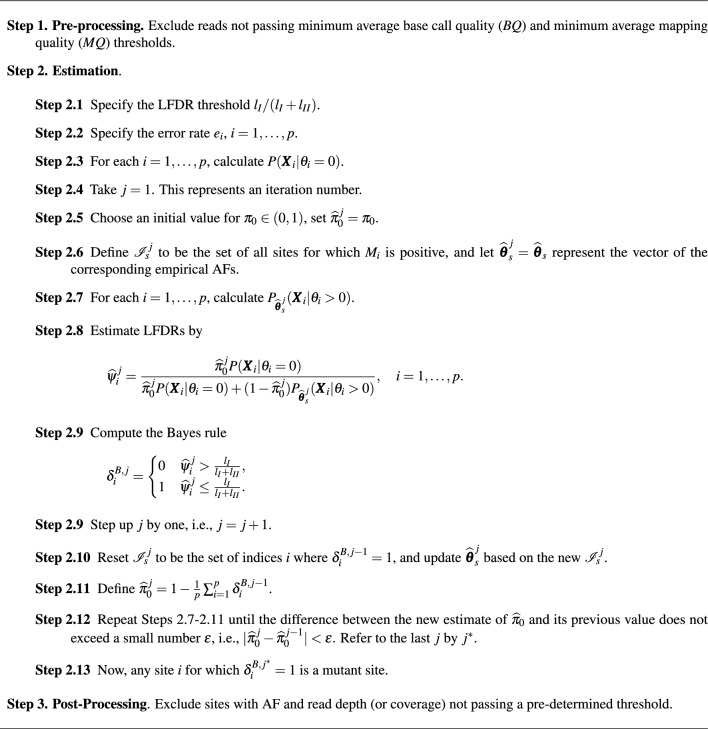
LFDR-based variant-calling algorithm.

### LFDR as a variant prioritization tool

We suggest that our LFDR calculation can be applied to prioritize any list of mutations called by any mutation caller from most to least probable mutations. This can be done by modifying Algorithm 1 so that $\widehat{\pi }_0$ is estimated as the number of reference sites according to the caller, out of all those assayed, and $g(\cdot )$ is estimated as the empirical distribution of variant sites. The LFDR calculation is then applied to all variant sites using these estimates of $\pi _0$ and $g(\cdot )$. Algorithm 2 outlines the prioritization steps.

**Algorithm 2:**
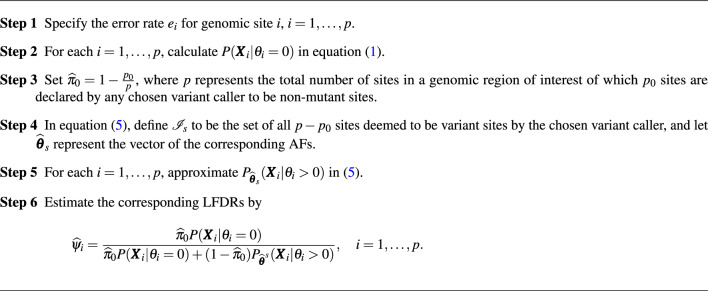
LFDR-based prioritizing algorithm for calls made by a desired variant caller.

## Results

We now focus on evaluating our proposed mutation calling algorithm on some data generated by clinical assays. We employ DNA sequencing data generated from two Coriell cell lines, GM12877 and GM12878, previously studied by Karimnezhad et al.^9^. These are immortalized healthy (non-cancer) cell lines that are expected to carry only germline variants. These cell lines have been extremely well characterized by numerous groups. Thus, we can know exactly which variants they harbor relative to the reference human genome, allowing us to accurately assess true and false positives and negatives. In particular, we rely on a list of known mutations published by Eberle et al.^22^ where a comprehensive and genome-wide catalog of high-confidence variants mutations for a collection of Coriell cell lines, including GM12877 and GM12878 is presented (available through https://www.ncbi.nlm.nih.gov/gap/ under accession phs001224.v1.p1). The data we used was sequenced in two different ways: (1) on an Illumina NextSeq500 sequencer using Illumina’s TruSight170 targeted sample preparation method (TST170 for short), and on an Ion Torrent PGM sequencer using an Oncomine Focus targeted panel (OF for short).

The TST170 data spans 514761 total bases, covering parts of 170 genes, and includes six technical replicates of each of the Coriell cell lines GM12877 and GM12878. By intersecting the list of known mutations with the TST170 genomic regions, we determined that our GM12877 and GM12878 data should contain 336 and 343 mutations, respectively. The OF panel covers 29008 total bases in 47 genes, and includes three technical replicates of each of the Coriell cell lines GM12877 and GM12878. There should be 24 and 26 known mutations present in each replicate of the GM12877 and GM12878 data, respectively. For more information regarding the datasets and also sequencing platforms, readers may refer to Karimnezhad et al.^9^.

For a given single replicate, denote the total number of true positives, false positives and false negatives by *TP*, *FP* and *FN* respectively. We measure the performance of the algorithms by computing precision or positive predictive value $Prec=\dfrac{TP}{TP+FP}$ and sensitivity $Sens=\dfrac{TP}{TP+FN}$. A good algorithm is expected to have high *Prec* and *Sens* rates.

To apply the algorithm on the TST170 as well as OF replicates, we used the bam-readcount package to calculate the counts $K_{i}$, $R_{i}$, $M_{i}$, $X_{1i}$ and $X_{2i}$, and then followed Steps 1-3 of Algorithm 1. We then compared the list of final detected variants with the lists of known mutations. Also, to investigate the impact of a chosen LFDR threshold in Step 2.1 of the algorithm on the detection accuracy, we picked $10^{-300}$, $10^{-200}$, $10^{-100}$, $10^{-50}$, $10^{-20}$, $10^{-10}$, $10^{-2}$ and 0.5.

We measured the performance of the proposed approaches by calculating *TP*, *FP*, *FN*, and consequently *Prec* and *Sens* values for different datasets and LFDR thresholds. As a second performance measurement, we compared the proposed algorithm with Mutect2, VarScan2, SAMtools, VarDict and Pisces. Illumina offers Pisces for the analysis of TST170 data through their customized pipeline. The application accepts paired-end fastq files as inputs, generates BAM files, and after aligning to the reference human genome (hg19) by the Isaac aligner^23^, uses Pisces to generate a list of mutations. Then, the final list of mutations is generated after some internal filtering. Our work was carried out in the context of a clinical sequencing project, where use of hg19 remains widespread, prompting us to use hg19 for alignments. However, we would expect very similar results with hg38. Our data is obtained by targeted sequencing of a panel of clinically-relevant genes, and there should be little or no difference between hg19 and hg38 for these highly-studied genes. We should add that, to compare the performance of our proposed algorithm with Pisces, as well as VarScan2, Mutect2, SAMtools and VarDict, we used the same aligned BAM files to reduce some possible alignment errors. Because differences in default parameters of the five algorithms in Table 1 are potential sources of discrepancies in the list of final mutations, we set their parameters as similar as possible. We set the minimum variant AF to 0.01. We set minimum base call quality and minimum mapping quality to 20. Finally, we set the minimum coverage for a called variant at 10 reads.

**Figure 2:**
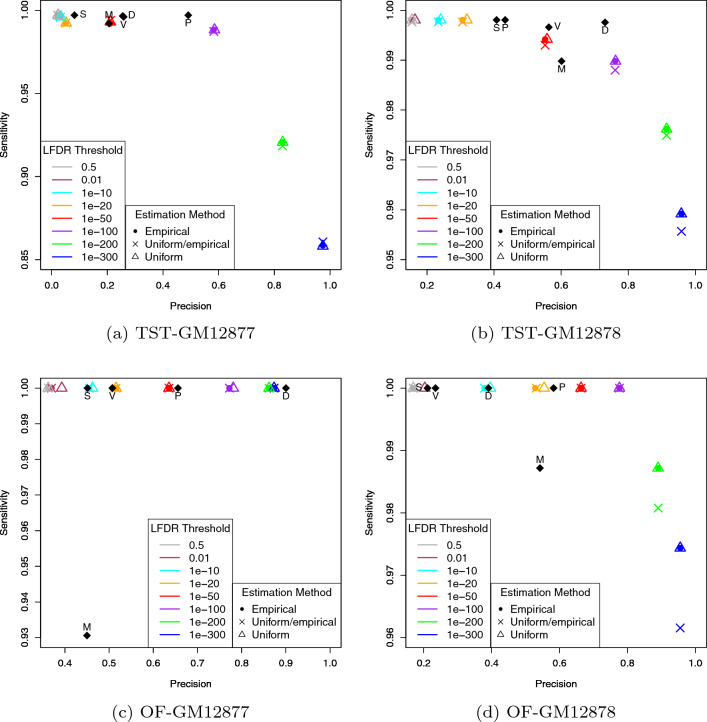
Average of *Prec* and *Sens* values over replicates based on calls made by the LFDR approach for different LFDR thresholds. Average of *Prec* and *Sens* values over replicates based on calls made by the other five variant callers were added for comparison purposes. M, V, S, D and P stand for MuTect2, VarScan2, SAMtools, VarDict and Pisces, respectively.

Figure 2 represents the average *Prec* and *Sens* values over replicates based on calls made by the LFDR approach for different thresholds, as well as the other five variant callers. From this figure, we observe that for all the LFDR thresholds, *Sens* values are mostly high while *Prec* is increased by decreasing the LFDR threshold. Indeed, when the LFDR threshold is high, say 0.5 for example, many false positives are allowed to be in the list of variants called. However, a strict LFDR threshold, say $10^{-50}$ for example, leads to few(er) false positives and consequently an improved *Prec* is gained. All the three estimation methods (empirical, uniform/empirical and uniform) performed nearly the same. Especially, when the LFDR threshold is below $10^{-50}$, the three methods led to almost the same *Prec* and *Sens* values.

**Figure 3:**
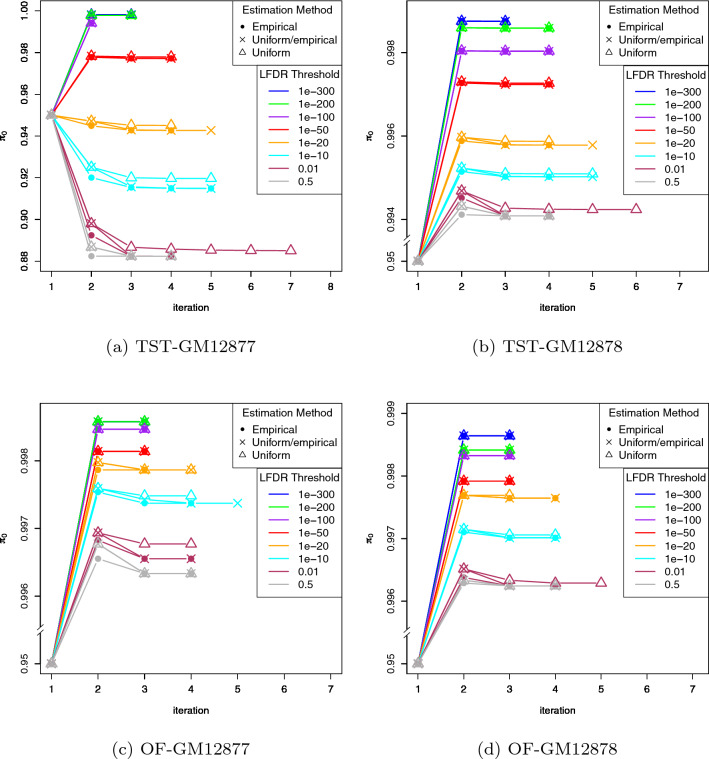
Estimated $\pi _0$ in replicate 1 of each dataset for different values of LFDR thresholds. The solid grey line represents the true $\pi _0$.

**Figure 4:**
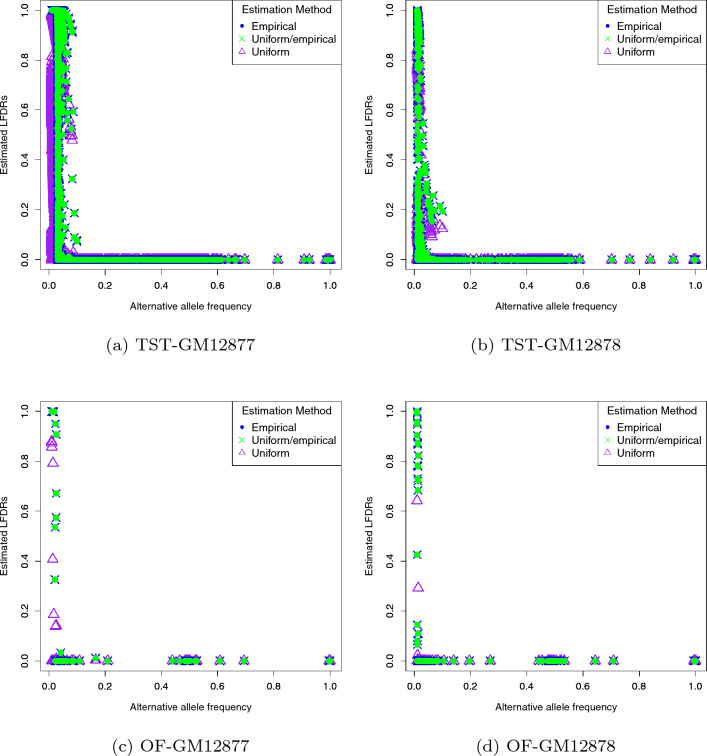
Estimated LFDRs for replicate 1 of each dataset based on taking $10^{-300}$ as the LFDR threshold.

Comparing with the other variant callers, we observe that the LFDR approach has equal or better *Sens* for some LFDR thresholds compared to all other algorithms. And for other thresholds, it has equal or better *Prec*. For example, from panel (a) in Fig. 2 we observe that the LFDR approach with a threshold of $10^{-100}$ outperforms all the other five variant callers in terms of *Prec*. However, it reports a smaller *Sens*. When looking at other panels, slightly different but remarkable performance is observed. For example, by choosing the same LFDR threshold, we observe in panel (b) that the LFDR approach outperforms MuTect2 in terms of *Prec*, and has almost the same *Sens*, and outperforms the remaining four variant callers in terms of *Prec*. However, its *Sens* is a bit smaller than the others. But when looking at panel (d), we observe that the LFDR approach with the same LFDR threshold outperforms all the other variant callers in terms of both *Prec* and *Sens*. We observe that having a varying LFDR threshold allows for a wide range of tradeoff between *Sens* and *Prec*.

Figure 3 represents convergence of estimated $\pi _0$ in replicate 1 of each dataset for different LFDR thresholds. From this figure, we observe that the algorithm converged mostly in 3-7 iterations, and most remarkably, for stringent LFDR thresholds ($10^{-100}$ or less), it converged in at most 4 iterations. We also note that in each panel, those stringent LFDR thresholds resulted in the greatest accuracy in estimating $\pi _0$. In fact, the lower is the LFDR threshold, the more accurate is the corresponding estimated $\pi _0$. Comparing with Fig. 2, we also observe that the most accurate estimated $\pi _0$ leads to the highest *Prec*. However, this is not neccessarily true when looking at *Sens* values.

We also investigated the impact of each method on the magnitude of LFDR estimates. Figure 4 represents estimated LFDRs for replicate 1 of each of our datasets based on taking $10^{-300}$ as the LFDR threshold. This figure reflects that both the empirical and uniform/empirical approaches coincide on detecting the same variants. The corresponding estimated LFDRs are mostly either close to zero (that corresponds to mutant sites) or one (that corresponds to non-mutant sites), revealing that both approaches were able to nicely categorize the sites as either mutant or non-mutant sites. But, LFDRs estimated using the uniform approach did not follow this structure and there are many sites with medium estimated LFDRs. This concludes that both the empirical and uniform/empirical approaches outperform the uniform approach in classifying sites to non-mutant as well mutant categories.

**Figure 5:**
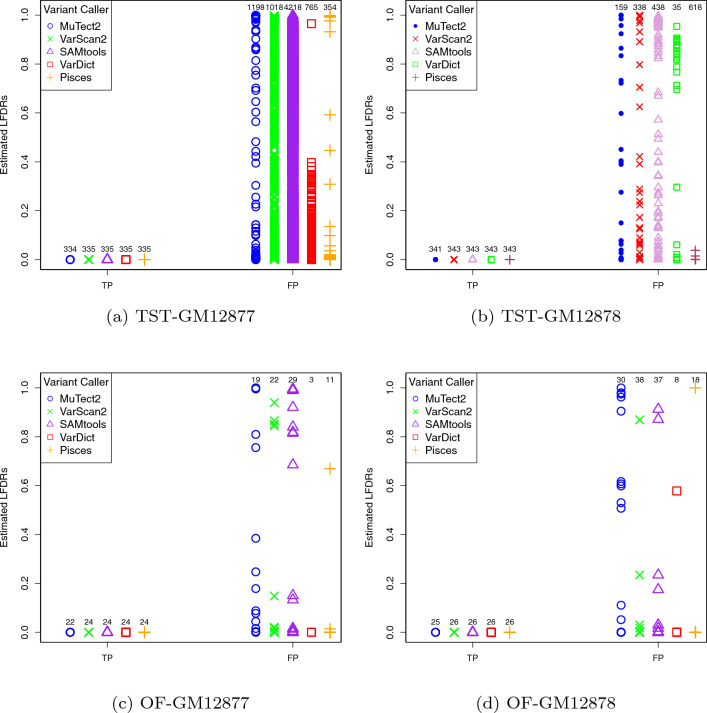
Estimated LFDRs for TP and FP calls made by the five varian callers in replicate 1 of each dataset.

Following the steps in Algorithm 2, we calculated the LFDR values for variants detected by the five variant callers (MuTect2, VarScan2, SAMtools, VarDict and Pisces) across replicate 1 of all the datasets. Figure 5 represents the corresponding LFDRs and whether the variants are TPs or FPs, along with the number of TPs and FPs. From the figure we observe that all those TPs led to either zero or very close to zero estimated LFDR values, as expected. For example, we observe from panel (a) that of the total 1532 variants detected by MuTect2, 334 variants with estimated LFDR values of zero or very close to zero are TPs, and 1198 variants with estimated LFDRs varying between zero and one are FPs. Comparing these numbers with 336, the number of known variants expected to be present in TST-GM12878 data, we realize that imposing a small LFDR threshold would dramatically reduce the number of FPs, and consequently *Prec* gets significantly improved. The lower is the LFDRs threshold, the more FPs are eliminated from the list of variants. Therefore, the proposed algorithm, with less complication compared to Algorithm 1, can prioritize results of other variant callers by simply eliminating FPs based on their LFDR values.

Naturally, prioritizing another variant caller’s output, and potentially deciding that some of those calls are below threshold, cannot increase *Sens*. *Sens* increase could only be achieved by adding in missed true variants. However, prioritization and thresholding has the potential to eliminate many FPs, and thus increase *Prec*, hopefully with little or no loss to *Sens*.

**Figure 6:**
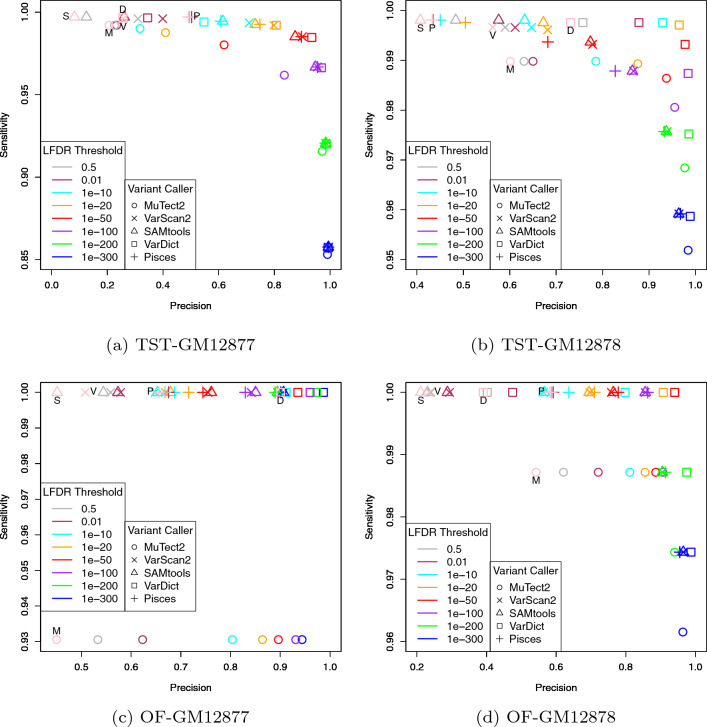
Average of *Prec* and *Sens* values for calls prioritized by the LFDR approach. The pink symbols refer to the *Prec* and *Sens* values of each individual variant caller calculated in Fig. 2.

We applied this prioritization approach to all calls made by the five variant callers in all replicates in the four datasets. We then calculated average *Prec* and *Sens* values for different LFDR thresholds as shown in Fig. 6. For comparison purposes, we also included *Prec* and *Sens* values calculated for each individual variant caller in Fig. 2. From Fig. 6, we observe that prioritizing variants using the LFDR method successfully leads to a significant increase in *Prec* values of the variant callers with minimal loss of *Sens* values for any threshold chosen between 0.5 and $10^{-50}$. As an example, from Fig. 6a, VarDict led to *Prec* and *Sens* values of 0.256 and 0.997, respectively, and prioritizing its calls using the LFDR approach with a threshold of $10^{-20}$, led to 0.801 and 0.992 as new *Prec* and *Sens* values. Thus, LFDR prioritization appears to be a powerful approach to further filter the output of other variant-calling algorithms.

## Discussion

In this paper, we introduced a novel LFDR-based approach that can be built into a SNV caller, or can be used to prioritize variants called by other algorithms. The algorithm requires some pre-determined numbers including the threshold $l_{I}/(l_{I}+l_{II})$ and an initial value for $\pi _0$. Although, the algorithm allows for introducing different error rates through $e_i$ at each genomic site to the model, users may set it to a global error rate $e=0.01$ which corresponds to a base call quality of 20. In fact, the default error value in some well performed software packages including Pisces^8^ has been set to 0.01. Assuming a global error rate facilitates the speed of computation and our data analyses convinced us that it has an ignorable effect on the number of detected mutations.

Both of our LFDR algorithms have few parameters to adjust and thus it is easier to tune, compared to the five variant callers we used in this study. Like the five variant callers we used, our algorithm uses some pre-specified numbers for *BQ*, *MQ* and error rates $e_i$, but it does not require any other parameter to adjust, except the LFDR threshold. And yet as an advantage, the threshold can be controlled by users/analysts and is easy to interpret, due to the fact that the LFDR is a probability and varies only on the interval [0, 1]. The closer to zero (one) it is, the more confidence is gained in detecting variant (non-variant) sites. We considered 8 different LFDR thresholds, and noticed that, thresholds between $10^{-100}$ and $10^{-20}$ lead to better results in term of *Prec* and *Sens* values on the datasets we studied. To establish the generality of this observation, further testing would be needed on other datasets from different sources or of different types.

We used three methods to estimate $g(\cdot )$ in Eq. (2). As expected, both the empirical and uniform/empirical approaches performed the same and identified the same variants. This is because both approaches lead to the same ${\mathscr {I}}_s$ set. The uniform approach also led to very similar results. This could be because we are ignoring the magnitude of LFDR values and we just compare them with the chosen thresholds. One possible explanation could also be the fact that the data we used in our project do not have any genuine mutations at low AFs, and thus, accurately estimating $g(\cdot )$ has little effect on the performance. However, as we showed in Fig. 4, the uniform approach is not able to firmly categorize sites to either mutant and non-mutants sites.

In this paper, we focused on germeline SNV calling. However, as the proposed model learns the form of $g(\cdot )$ in Eq. (2), the proposed SNV calling approach may be used in a tumor-only mode, without comparison to healthy tissue from the same patient, which is often the case in clinical sequencing. The accuracy of the proposed method for tumore-only samples remains open to be investigated.

One may incorporate uncertainty about the parameter $\theta _i$ using other Bayesian framewroks. For example, one may compute the Bayes factor $BF=\frac{P({\varvec{X}}_i | \theta _i=0)}{P({\varvec{X}}_i | \theta _i>0)}$ and re-express the LFDR quantity defined in Eq. (3) as

$$\begin{aligned} \psi _{i}=\frac{\pi _{0}BF}{\pi _{0}BF +(1-\pi _{0})}, \end{aligned}$$

or

$$\begin{aligned} \psi _{i}=\frac{PO\times BF}{PO\times BF +1}, \end{aligned}$$

where $PO=\frac{\pi _0}{1-\pi _0}$ can be interpreted as the prior odds of having no mutations, see Wakefield^21^ for more details. However, having the BF alone does not seem to be enough to identify variant sites and $\pi _0$ still needs to be somehow estimated. The beauty of our proposed approach is that it estimates both $P({\varvec{X}}_i | \theta _i>0)$ (and hence the Bayes factor) and $\pi _0$ without having any prior information regarding the distribution of $\theta _i$.

We end our discussion with highlighting that in some situations, one may notice conflicts in calling a site a variant site. This may happen when multiple replicates are studied simultaneously and a variant caller detects a mutation at a specific site in one replicate and does not call that variant in the other replicate(s). One simple way to resolve this inconsistency would be to take the intersection of the variants called in all replicates. However, this may exclude some important variants (if borderline) and in general ignores the confidence any one replicate gives us. In an extreme case, if a replicate failed to have data in a region, and thus no variants were called, that lack of data would effectively overrule the positive data in other replicates. Alternatively, one may seek to take advantage of advanced decision-theoretic approaches. In a different but still applicable context, Karimnezhad and Bickel^19^ developed an empirical Bayesian approach that takes advantage of prior information from multiple reference classes and leads to a unique decision in conflicting situations. This could be a potential future research problem.

## Data Availability

All sequencing data is available from the Sequence Read Archive under project accession PRJNA614006. Human genome version hg19/GRCh37 is available from the UCSC Genome Browser website (genome.ucsc.edu).

## Code availability

The R code implementing the proposed methods is available on Github https://github.com/empiricalbayes/SNVLFDR.
